# The profile of microorganisms that associate with acute tonsillitis in children and their antibiotics sensitivity pattern in Nigeria

**DOI:** 10.1038/s41598-021-99570-9

**Published:** 2021-10-11

**Authors:** Maduka Donatus Ughasoro, James Onuorah Akpeh, Nneamaka Echendu, Nneka Gertrude Mgbachi, Somkene Okpala, Linda Amah, Onyinye Henrietta Okolo, Ngozika Udem

**Affiliations:** 1 Department of Paediatrics, University of Nigeria Enugu Campus, Enugu, Nigeria; 2 Department of Otorhinolaryngology, University of Nigeria Enugu Campus, Enugu, Nigeria; 3 Department of Paediatrics, University of Nigeria Teaching Hospital, Ituku/Ozalla, Enugu Nigeria; 4 Department of Microbiology, University of Nigeria Teaching Hospital, Ituku/Ozalla, Enugu Nigeria; 5 Department of Paediatrics, Federal Medical Center, Umuahia, Abia State Nigeria; 6 Pharmacy Department, University of Nigeria Teaching Hospital, Ituku/Ozalla, Enugu Nigeria

**Keywords:** Microbiology, Diseases, Medical research

## Abstract

Acute tonsillitis remains one of the common childhood diseases in developing countries. Prompt and appropriate treatment based on the knowledge of the causative microbiota and their antimicrobial susceptibility pattern will improve the treatment outcome and reduce time and resources spent on treatment. This study aims to determine the pattern of microbiota isolates and their susceptibility pattern. The study was a combination of the retrospective and cross-sectional method. The medical records of children treated for tonsillitis were retrieved, as well as those of children who presented with acute tonsillitis for the study. Interviewer-administered questionnaire was used to collect data, as well as document information retrieved from their medical record: presenting symptoms, treatments received. Swab sample was taken for culture and antibiotic susceptibility test. Out of the 72 swab cultures, 29 (40.3%) yielded insignificant growth of gram positive cocci. A total of nine (9) different isolates were obtained from all the significant swab cultures. Streptococcus species (13, 18.6%) and staphylococcus species (11, 15.3%) were the commonest isolates. Imipenem and levofloxacin revealed high level of susceptibility, while Ampicillin and Cefexine recorded high resistance rates. The proportion of the cultures that were positive of significant growth, the proportion of these positive isolates that were *Streptococcus* spp. and varied sensitivity pattern obtained underpinned the importance to advocate for culture isolates and susceptibility pattern guided treatment. These will not only an efficient approach to management of acute tonsillitis, but also a strong approach towards effective implementation of antibiotic stewardship.

## Introduction

Acute tonsillitis in children is an extensive inflammatory upper respiratory tract infection associated with the inflammation of the lymphoid tissue of the palatine tonsillar. It is also referred to as tonsillopharyngitis, meaning essentially lesion of the oropharyngeal lymphoid ring and not restricted to only the tonsils. When the infection has lasted for just a short period of time it is acute tonsillitis^1^. Acute tonsillitis is uncommon in children under 1–1.5 years of age, because the maturation and completion of the tonsillar ring’s lymphoid tissue occurs after that age. The etiology in children under 3–4 years of age, are mainly viral or virobacterial etiology (adenoviruses, Coxsackie enteroviruses etc.) in about 95% of cases. The group A β-hemolytic streptococcus (*Streptococcus pyogenes*, GABHS) becomes significant in the causative organisms of acute tonsillitis in children over 4 years of age until the age of 18 years^2,3^. Recently, conjugated pneumococcal vaccine was introduced in the routine immunization programme of Nigeria. This vaccine promised to cover for the *Streptococcal pneumonia* which causes most of the upper respiratory tract infections and pneumonia.

Timely and correct diagnosis of streptococcal tonsillitis and further adequate antibacterial treatment can lead to prevention of acute rheumatic fever and purulent complications^4^, as well as renal complications. Like in other disease control programme, the laboratory confirmation of disease condition before treatment is pivotal^5–8^, and feasibility if rapid diagnostic test (RDT) is available^9,10^. But for diseases like Acute Tonsillitis which can be caused by multiple organisms, the lack of rapid diagnostic kit for these common organisms makes implementation of prompt laboratory diagnostic difficult especially in resource poor countries.

Culture examination of the ill child’s oropharyngeal swab can reveal the etiologic agent of tonsillitis and it is the gold standard. Systemic antibacterial therapy is most appropriate in the treatment of complicated tonsillitis especially with abscess, and evidence of streptococcal etiology of the inflammatory process^11^. Unfortunately, it is not a common practice in the African sub-region to wait for culture isolates and antibiogram before commencing antibiotic treatment. With the current ease in excluding malaria cases with MRDT^12,13^, and the ease of clinically detecting inflamed, enlarged and exudative tonsils or discharging ears, the empirical treatment of tonsillitis, using antibiotics stands to increase. Due to the abundant sensitive innervation of the pharyngeal mucosa, any pathological processes are accompanied with barrage of symptoms include pain^14^ and paediatricians and otorhynolaryngologists are compelled to treat empirically^15,16^. Despite evidence that microorganisms that causes acute tonsillitis are constantly changing both as isolates and even within isolates, their susceptibility to antibiotics varies^17–19^.

Profiling of isolates and their antibiotic susceptibility, will guide decision to treat and the choice of empirical antibiotics to use. This study aims to determine the prevalence of bacterial isolates in the childhood tonsillitis, and their antibiotic susceptibility.

## Methods

### Study area and population

The study was conducted in the children outpatient clinics of University of Nigeria Teaching Hospital, Ituku-Ozalla, Enugu State, and Federal Medical Center, Umuahia, Abia State, both in Southeast Nigeria. The UNTH, is a tertiary health facility located at Ituku/Ozalla, Enugu State, Southeast Nigeria. Enugu State has a population of approximately 3.3 million people^20^, according to national census of 2006. The children under 14 years make up 41 percent of the entire population^21^. There are laboratories that carry out different investigations and pharmacy that dispense drugs.

Federal medical Center, Umuahia is a tertiary health institution located in Umuahia, the capital of Abia state. The children out-patient clinic of the hospital renders service 5 days in a week. The hospital has well-equipped laboratories, among which is a microbiology laboratory.

### Study design

The study was a combination of both retrospective and cross-sectional approach on cases of acute tonsillitis who presented to the clinic recruited.

### Sample size calculation

The Epi-Info software was used to calculate the sample size. The Epi-Info version 7^22^ was used to estimate the minimum sample size. This was based on the input of the prevalence of childhood tonsillitis of 11%^23^ and 95% confidence interval and power of 80%. The subjects were consecutively recruited as they attend the clinics. The sample size were calculated using the formula for bioequivalents and prevalence of acute tonsillits of 11%. N = Z^2^ *p*(1 – *p*)/*d*^2^ where N = is the size of sample for each group of treatment, Z^2^ is standard normal variate at 5% type 1 error (*P* < 0.05) and is equivalent to = 1.96, *p* = expected proportion in the population based on previous study of prevalence of 11%, d = absolute error or precision of 5% (0.05). Therefore, N = (1.96)^2^ * 0.11(1 – 0.11)/(0.05)^2^ = 150 children with acute tonsillitis. To recruit the sample size, all the children that present to children out-patient clinics with fever, whose caregiver gave consent to be part of the study were recruited. A total of 172 children with tonsillitis were recruited. The questionnaire was pre-tested and, ambiguous questions modified for clarity.

### Data collection

The medical records of children managed for either tonsillitis over the period of 5 years from January 2014 to December, 2019 were retrieved and reviewed. The information on their age, gender, place of resident, household sizes, presenting symptoms, health seeking behaviors and treatment received were documented in a questionnaire. Children with enlarged, inflamed and exudative tonsils, and or ear discharge, who presented to children out-patient clinics from March 2019 to September 2019 had their throat swab taken for microscopy, culture and sensitivity.

### Sample collection

The oral cavity of the child was opened widely, illuminated adequately with a head lamp, and the tongue depressed with a sterile wooden specular. The sterile swab stick was rubbed firmly on the surfaces of the inflamed tonsils, avoiding contact with the tongue or buccal mucosal.

### Sample culture and identification

Samples were cultured on Chocolate agar, Blood agar and MacConkey agar plates by streaking method and incubated at 37 °C for 24 h aerobically and in CO_2_ reduced atmosphere. The plates were then examined for microbial growth. All isolates were identified using their growth morphology on culture media, haemolysis, Gram’s staining, and motility test and biochemical identification. All Gram negative rods were further identified biochemically on Lactose fermentation, Oxidase utilization, Catalase, Methyl red, Indole, Citrate, Urease, Nitrate reduction, Pigmentation, H2S production, Gelatin hydrolysis and capsule formation. Likewise, all isolates that were Gram positive were further identified on Haemolysis, Spore formation, Catalase, Coagulase, CAMP test, Bile solubility, Optochin sensitivity and Bacitracin sensitivity as in Cheesebrough et al.^24^.

### Sensitivity test

The antibiotic Sensitivity tests were performed on the pathogenic isolates by Kirby Bauer disc diffusion technique using 0.5 McFarland standard. This was done using Muellar Hinton agar media^17,25,26^. Using aseptic technique, 3–5 discrete colonies of the microbial growth were suspended in about 2 ml of sterile normal saline and vortex to obtain smooth suspension. The turbidity was adjusted to 0.5 McFarland standard. Using cotton tipped sterile swab stick the surface of the Mueller Hinton agar was inoculated. Sterile forceps were used to manually place the antibiotic discs on the culture plates and incubated at 37 °C for 18 h. The zones of inhibition were measured using metric ruler a few inches above a black non-reflecting surface and measurements were compared with standard tables using the Committee for Clinical Laboratory Standards Institute^26^. The choice of antibiotics was based on commonly used antibiotics obtained from review of medical records and the feasible combination available in the antibiogram kit. Sensitivity was based on observed standard clearance around the antibiotic disc^26^. Direct microscopy was done using the isolates from the culture media^27^.

The culture and sensitivity was done at the Microbiology laboratory of University of Nigeria Teaching Hospital, Ituku-Ozalla, Enugu. The samples collected from Federal Medical Center, Umuahia, were put in ice pack and later refrigerated and sent in batches every 2 days (Wednesday and Fridays). Samples were not collected on Fridays, Saturdays and Sunday.

### Data analysis

The data was entered and analyzed using SPSS version 20. Frequencies were calculated. Yates correction was used in variables with less than value of 0.05 to determine the significant.

### Ethical consideration

The approval for the study was obtained from Health Research and Ethics Committee of University of Nigeria Teaching Hospital, Ituku-Ozalla. The Ethical Clearance Certificate number was NHREC/05/01/2008B-FWA00002458-1RB00002323. Issued on 1^st^ April 2019. The Institutional reference number was UNTH/CSA/329/OL.5. All methods were performed in accordance with the relevant guidelines and regulations as described in the Helsinki Declaration. Information about the study was explained to the parents/caregivers of these children and written informed consent was obtained before recruiting them into the study. The results of the swab microscopy and sensitivity were held in confidentiality. The children were commenced on empirical antibiotics, which were later reviewed with the sensitivity results for the positive isolates.

### Consent to participate

Written informed consent was obtained from all participants involved in the study. Information about the study was explained to the parents/caregivers of these children. The results of the swab microscopy and sensitivity were held in confidentiality. The children were commenced on empirical antibiotics, which were later reviewed with the sensitivity results for the positive isolates.

## Results

There were 91 (52.9%) males involved in the study. Their median age was 2 years. Their median duration of onset of symptoms was 4 days. Majority 124 (72.1%) sought for care before presenting to the hospital. See Table 1.

**Table 1:** Sociodemographic characteristics of the subjects.

Variables	n	%
**Gender (n = 172)**		
Male	91	52.9
Female	81	47.1
**Age (in years)**		
Mean	3.8 years	
Median	2 years	
**Duration of illness (in days)**		
Median	4 days	
**Sought for care (n = 172)**		
Yes	124	72.1%
No	48	27.9%
**Previous episode(s) of Tonsillitis**		
Yes	24	14%
No	148	86%
**Informant (n = 172)**		
Mother	151	87.60%
Father	11	6.20%
Grandmother	4	2.30%
Aunt	2	1.20%
Others	5	2.70%
**Mothers' education (n = 151)**		
None	0	0%
Primary	2	1.30%
Secondary	26	17.20%
Tertiary	123	81.50%
**Mothers' occupation (n = 144)**		
Civil servant	45	31.30%
Business	25	17.40%
Housewife/unemployed	23	16%
Teacher	19	13.20%
Nurse	7	4.80%
Petty trader	4	2.80%
Others	21	14.50%

The proportion of the subjects that received antibiotics and antimalarial before presenting to the hospital were about 47 (35.6%) and 51 (38.7%) respectively. Cough a respiratory system symptom and vomiting a digestive system symptom were the commonest with prevalence of 74 (43%) and 35 (20.3%) respectively. See Table 2.

**Table 2:** The common medication taken before presentation and the presenting symptoms of the subjects.

Pre-medications	n = 132	%	Presenting symptoms	n = 172	%
**Antibiotics**			**General**		
Amoxicilli/Ampicillin	21	15.90%	Fever	127	73.80%
Augumentin/Co-amoxiclav	26	19.70%	Weakness	7	4.10%
Azithromycin	3	2.30%	**Respiratory**		
Cefexine	2	1.50%	Cough	74	43%
Ceftriaxone	2	1.50%	Catarrh	45	26.20%
Cefuroxine	4	3%	Ear Pain	12	7%
Ciprofloxacin	1	0.8	Ear discharge	12	7%
Cotrimoxazole/Septrin	4	3%	Noisy breathing	11	6.40%
Erythromycin	5	3.90%	Sore throat	9	5.20%
Metronidazole	4	3%	Mouth breathing	4	2.30%
Orelox/Cefpodoxine	7	5.30%	Difficult breathing	3	1.70%
**Antimalarial**			**Digestive**		
ACTs	41	31.10%	Vomiting	35	20.30%
Artemisinin Monotherapy	10	7.60%	Throat pain	27	15.70%
Camoquine	3	2.30%	Loose stool	17	9.90%
Chloroquine	1	0.00%	Loss of appetite	14	8.10%
			Abdominal pain	8	4.70%
			Salivation	3	1.70%
			Mouth dour	1	0.60%
**Antipyretics**			**Central nervous**		
Ibuprofen	19	14.40%	Convulsion	6	3.50%
Paracetamol	34	25.80%	Headache	5	2.90%
			Excessive cry	1	0.60%
**Other medications**			**Others**		
Cough syrup	18	13.60%	Pains	10	5.80%
Vitamin C	6	4.50%	Rash	5	2.90%
Astymin	3	2.30%	Swelling	2	1.20%
Multivite	1	0.80%	Eye discharge	1	0.60%

**Figure 1:**
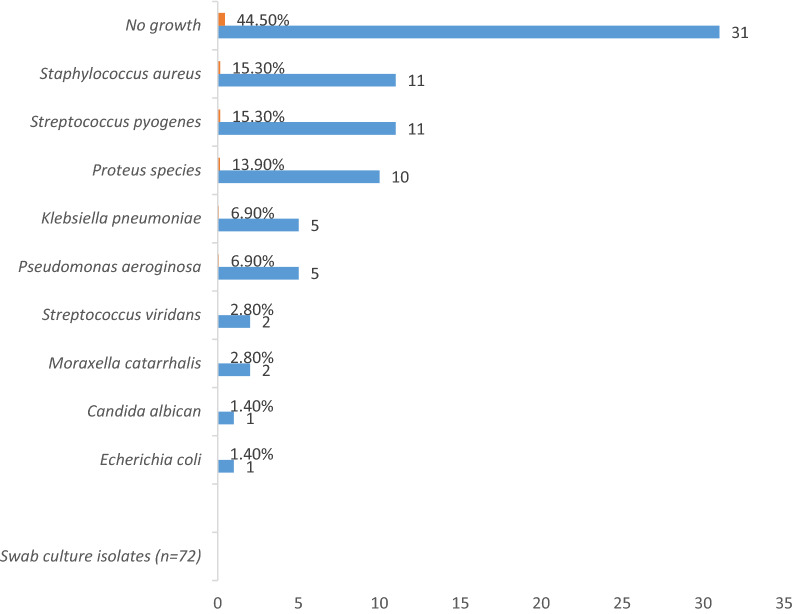
The microorganism isolates from the swab culture.

Most 29 (40.3%) of the swab culture yielded insignificant growth of gram positive cocci. A total of nine (9) different isolates were obtained from the swab culture. Among the significant isolates were *streptococcus species* (13, 18.6%) and *staphylococcus species* (11, 15.3%). See Fig. 1.

Imipenem (45, 95.7%) and Levofloxacin (37, 78.7%) had the highest sensitivity across all the isolates put together. Ceftriaxone, Azithromycin, and Amoxicillin/Clavulanic acid had sensitivity of 68.1% (32), 57.4% (27) and 42.6% (20) respectively. The least sensitive antibiotics were ampicillin 8 (17%) and Cefexine 17 (36.3%). See Table 3.

**Table 3:** The Antibiogram susceptibility and resistant pattern of the microbiota isolates.

Isolates	Sensitivity pattern	Imipenem	Levofloxacin	Ceftriaxone	Azithromycin	Amoxicillin-Clavulanic	Cefexine	Ampicillin
*Staphylococcus aureus* (n = 11)	Sensitive	9	11	7	6	8	8	4
	Resistant	2	0	4	5	3	3	7
*Streptococcus species* (n = 13)	Sensitive	13	11	13	9	5	4	2
	Resistant	0	2	0	4	8	9	11
*Proteus* (n = 10)	Sensitive	10	7	3	5	2	2	0
	Resistant	0	3	7	5	8	8	10
*Klebsiella* (n = 5)	Sensitive	5	3	3	3	0	0	0
	Resistant	0	2	2	2	5	5	5
*Pseudomonas* (n = 5)	Sensitive	5	4	3	1	2	2	1
	Resistant	0	1	2	4	3	3	4
*Morexella catalalis* (n = 2)	Sensitive	2	0	2	2	2	0	0
	Resistant	0	2	0	0	0	2	2
*E. coli* (n = 1)	Sensitive	1	1	1	1	1	1	1
	Resistant	0	0	0	0	0	0	0
	Summed sensitivity	45 (95.7%)	37 (78.7%)	32 (68.1%)	27 (57.4%)	20 (42.6%)	17 (36.3%)	8 (17%)
	Summed resistant	2	10	15	20	27	30	39

Amoxicillin/Clavulanic acid (26, 19.7%) and Amoxicillin or Ampicillin (21, 15.9%) were the commonly used drugs before hospital presentation. See Table 2.

The proportion of paediatric who presented with the first episode of tonsillitis with positive bacterial isolates were higher (44, 29.7%) than in those with recurrent episode(s) of tonsillitis (3, 12.5%), but it was not a statistically significant difference (*p *value = 0.079). See Table 4.

**Table 4:** Comparative analysis of the isolates between the 1st episodes and those with previous episodes.

	Episodes of tonsillitis		*χ*^2^	*p *value
	First episode	Had previous episode(s)		
Positive bacterial isolate(s)	44 (29.7%)	3 (12.5%)	3.087	0.079
No bacterial isolate(s)	104 (70.3%)	21 (87.5%)		

## Discussion

Majority 72.1%, of the parents/caregivers of these paediatrics with acute tonsillitis sought for care prior to presenting to tertiary health facility. It is a common practice for parents to try some medications which can be remaining drugs used for treatment in the previous illnesses^28^, or purchased from the drug retailers in the course of the present illness^29^. Among the major reason for seeking for health are symptoms suggestive of malaria, acute respiratory infections, and diarrhea as reported by Adegboyega et al.^30^ Most only seek for care from health facilities when the expected relief from self-treatment was not realized. This delay might explain the median duration of illness of 4 days obtained in this study. Extensive behavioral communication change campaign targeted towards mothers, should be deployed through all the contacts and avenues, as well as interventions that can reduce the barriers to access and utilization of services of healthcare facilities^31^.

Antibiotics and antimalarial usage prior to presentation were high. This practice has a dire consequences since studies have shown that varied proportion of treatment administered both at home and at facilities were faulty either in dosage or in timing^32^. This poor antibiotic practices could contribute to the poor susceptibility of the isolates to the commonly used antibiotics, which about a decade ago recorded good susceptibility to the same isolates^33^. In the study by Sadoh et al.^33^, Beta Hemolytic *Streptococcus* and *Staphylococcus aureus* showed 100% susceptibility to azithromycin, but in this study, the same organisms showed 70% and 54.5% respectively. A loss in sensitivity of 30% and 46.5% respectively in an interval of 14 years. Although Imipenem and Levofloxacin showed a good susceptibility. These are relatively new drugs, imipenem has no oral preparations and levofloxacin is not a commonly used drugs, so difficult to abuse in the community. Furthermore, some commonly used non-anti-psuedomonal drug like Azithromycin was included in the sensitivity because it is among the antibiotics commonly used in treatment of upper respiratory tract infection^33–37^. This observed change is of great concern with the new WHO report that very few antibiotics currently in development address the serious and growing threat of antimicrobial resistance to classes of priority pathogens identified by World Health Organization, according to the Global Antimicrobial Resistance Surveillance System (GLASS)^38^.

About 42.5% of the swab culture yielded no growth or insignificant growth of gram-positive cocci. This finding was similar to other studies that reported no isolates ranging from 46.6 to 75.6%^33,39^. This variation in culture yield could be attributed to the findings in a review by van der Veen et al.^40^ In their work, they found that a higher positive culture results (55%) were found in the swab taken from the posterior pharyngeal wall compared to 35% obtained from the swab taken from the tonsillar surface among children selected for adenotonsilectomy. Furthermore, it has been shown that variations in the microbial flora do essentially play a distinct role in the predisposition of children to tonsillar disease^41^. The proportion of isolates were Enterobacterieceae. The Enterobacterieceae is a large group of Gram-negative bacteria that includes pathogens like *Klebsiella, Proteus* and *Escherichia coli* among others*,* which is a normal part of the human intestinal tract (gut) flora, deserve explanation. Similarly, other studies have isolated these organisms from the oro-pharynx were among paediatrics^42,43^. This could be as a result of certain habits like; premastication of meat and other foods by adults for their children^44^ and general poor hygiene could predispose to being infected by these organisms. Thus clinicians need to evaluate the risks and benefits prior to prescribing immediate antibiotics for uncomplicated tonsillitis. History of recurrent episodes of acute tonsillitis, which is often the reason for performing surgical procedures in children, did not show any statistically significant difference in the prediction of positive swab culture isolate(s). Therefore each child should be evaluated individually, reviewing the presenting complaints, with swab investigation result, before prescribing antibiotic if it will be needed.

One envisaged challenge is the healthcare providers being under pressure to commence empirical treatment without knowledge on investigation result. In view of some challenges: laboratory result taking an average of 3 days for results to be ready, and parents travelled some distance to present to the health facility and will be anticipating some form of treatment. But study has shown that caregivers are willing to do investigation if prescribed by a physician^45^. Paediatricians and Ortorynolaryngologists need to realize that most of the parents/caregivers opt to use formal health care facilities when they have experienced failure with their previous self-medication. In this study, 72.1% of the parents/caregivers had received some form of treatment before presenting. Therefore, at the point they came for care, they are highly willing to get relief of their children symptoms. Notwithstanding, this claim, majority of the healthcare providers have the strong perception that parents/caregivers would expect their ill children to be commenced on treatment while awaiting for the outcome of the investigation, as noted by Britten et al.^46^ All parents/caregivers want prescriptions, basically on their anticipation of what will improve the health condition of their sick child, not particularly the content of the prescription. Therefore, healthcare providers can commence the child on symptomatic treatment/agents consist of topical preparations (slowly disintegrating tablets, sprays, lozenges, antiseptic containing mouthwashes like chlorhexidine, anesthetics like lidocaine, antibiotics, vitamins especially ascorbic acid and deodorants^14^. Only the most efficient and safe drugs will be recommended for children with uncomplicated cases on an out-patient basis until their swab test result is established. For patients with features of complications like abscess, they can be admitted on supportive care and for surgical evaluation, until the isolate(s) is/are confirmed and susceptibility pattern known. It therefore means that with appropriate supportive care, commencement of antibiotic treatment can be withhold for a while or rather till it is necessary. The delay in commencing definitive antibiotic treatment can be reduced if not entirely eliminated with deployment of group A Beta haemolytic streptococci rapid test kits^47^. The concern on default to follow if the child improved the symptomatic treatment does not really hold, since it has been shown that respondents are willing to return for the result of their tests and also for addition of more medication at a later date, even if their children have made remarkable improvement^45^. Efforts should also be made to explain treatment plan, so that in the event that their children improved on supportive care, they would not be compelled to carry over the same treatment in future re-occurrence of similar symptom(s). Considering the close similarity the symptoms of tonsillitis have with other illnesses that may be more sinister, as well as the complications of ill-managed streptococci tonsillitis: rheumatic fever, and renal complications^48,49^.

## Limitations

This study has one major limitation that needs to be addressed in subsequent study. The inclusion of 47 (35.6%) of patients that were on antibiotics prior to the presentation and the culture has potential of affecting the interpretation of the results. However, if this group was dispersed in the entire subjects, it may reduce the percentage of positive yield but not the proportion of the cultured organisms. Although the persistence of symptoms and inflammation as at the time of presentation is an indication that the causative microbes are still present and active. Going forward future studies may require two separate groups: a group not on antibiotics and a second group of patients already on antibiotics, to be reported on. Another limitation is that the entire sample size of 172 children with acute tonsillitis recruited for the study, not all had throat swab taken for microscopy, culture and sensitivity, due to limited fund available for the study, and thus can affect the generalization of the findings to entire population of children with acute tonsillitis. However, the scope of the micro organisms isolated and their sensitivity pattern may not vary widely if large population survey was carried, but the prevalence of isolation of each of the micro organisms may vary. Therefore, a larger population survey may be required to establish this fact.

## Conclusion

The proportion of the cultures that were positive of significant growth, the proportion of these positive isolates that were *Streptococcus* spp. and varied sensitivity pattern obtained underpinned the important to advocate for culture isolates and susceptibility pattern guided treatment. This is not only an efficient approach to management of acute tonsillitis, but also a strong approach towards effective implementation of antibiotic stewardship.

## Data Availability

More complete data are available from the authors upon request.
